# Desensitization of cAMP Accumulation via Human β3-Adrenoceptors Expressed in Human Embryonic Kidney Cells by Full, Partial, and Biased Agonists

**DOI:** 10.3389/fphar.2019.00596

**Published:** 2019-06-07

**Authors:** Katerina Okeke, Martina B. Michel-Reher, Stavros Gravas, Martin C. Michel

**Affiliations:** ^1^Department of Pharmacology, Johannes Gutenberg University Mainz, Mainz, Germany; ^2^Department of Urology, University of Thessaly, Larissa, Greece

**Keywords:** β3-adrenoceptor, cAMP, extracellular signal-related kinase, desensitization, partial agonism, biased agonism

## Abstract

β_3_-Adrenoceptors couple not only to cAMP formation but, at least in some cell types, also to alternative signaling pathways such as phosphorylation of extracellular signal-regulated kinase (ERK). β_3_-Adrenoceptor agonists are used in long-term symptomatic treatment of the overactive bladder syndrome; it is only poorly understood which signaling pathway mediates the clinical response and whether it undergoes agonist-induced desensitization. Therefore, we used human embryonic kidney cells stably transfected with human β_3_-adrenoceptors to compare coupling of ligands with various degrees of efficacy, including biased agonists, to cAMP formation and ERK phosphorylation, particularly regarding desensitization. Ligands stimulated cAMP formation with a numerical rank order of isoprenaline ≥ L 755,507 ≥ CL 316,243 > solabegron > SR 59,230 > L 748,337. Except for the weakest agonist, L 748,337, pretreatment with any ligand reduced cAMP responses to freshly added isoprenaline or forskolin to a similar extent. On the other hand, we were unable to detect ERK phosphorylation despite testing a wide variation of conditions. We conclude that a minor degree of efficacy for cAMP formation may be sufficient to induced full desensitization of that response. Transfected human embryonic kidney cells are not suitable to study desensitization of ERK phosphorylation by β_3_-adrenoceptor stimulation.

## Introduction

β_3_-Adrenoceptors have a restricted expression pattern in humans (Michel and Gravas, 2016) but are the main receptor mediating relaxation of human urinary bladder smooth muscle (Michel and Vrydag, 2006). Therefore, β_3_-adrenoceptor agonists have become an option for the treatment of patients with overactive bladder syndrome (Ohlstein et al., 2012; Chapple et al., 2014; Yoshida et al., 2018). Such treatment effectively relieves symptoms but is not curative, implying that it requires chronic and possibly life-long use. G protein-coupled receptors (GPCRs), including β_1_- and β_2_-adrenoceptors, typically undergo agonist-induced desensitization for instance in the treatment of heart failure (Mauro and Mauro, 1986) or pre-term labor (The Canadian Preterm Labor Investigators Group, 1992), respectively. Therefore, the question arises whether agonist-induced desensitization of β_3_-adrenoceptors may become treatment-limiting.

β_3_-Adrenoceptors were initially thought to be resistant to agonist-induced desensitization because of a relative lack of consensus phosphorylation sites in the C terminus as well as the tyrosine residues in the cytoplasmic loops of the receptor (Emorine et al., 1989) that are deemed critical for agonist-induced desensitization in other GPCR. Indeed, cAMP formation upon β_3_-adrenoceptor stimulation has been found to lack agonist-induced desensitization in multiple cell types natively expressing the receptor including rat adipocytes (Granneman, 1992) and rat cardiomyocytes (Germack and Dickenson, 2006); it was also reported to be absent in some cell lines transfected with the human β_3_-adrenoceptor including Ltk^−^ and CHW cells (Nantel et al., 1993). On the other hand, agonist-induced desensitization of β_3_-adrenoceptor function and/or down-regulation of receptor mRNA and/or protein has been observed in multiple tissues and cell types endogenously expressing β_3_-adrenoceptors including mouse ileum, rat and mouse white and brown adipose tissue, hamster and mouse brown adipocytes, the murine 3T3-F442 adipocyte-like cell line, and SK-N-MC human neuroblastoma cells (Okeke et al., 2019). Moreover, desensitization of cAMP formation has consistently been observed in human embryonic kidney (HEK) cells transfected with the human or rat β_3_-adrenoceptor (Chaudhry and Granneman, 1994; Vrydag et al., 2009; Michel-Reher and Michel, 2013). Agonist-induced desensitization of cAMP formation in this model is time- and concentration-dependent and largely consists of a reduced maximum effect; in contrast, there is little effect on agonist potency or receptor protein density, and desensitization does not depend on polymorphisms of the receptor or changes of G-protein expression.

While the canonical signaling pathway of β_3_-adrenoceptors is coupling to G_s_ proteins to stimulate adenylyl cyclase, they can also couple to additional signaling pathways including inhibition of adenylyl cyclase, stimulation of extracellular signal-regulated kinase (ERK) (Gerhardt et al., 1999; Soeder et al., 1999; Cao et al., 2000; Sato et al., 2008), p38 mitogen-activated protein kinase (Mizuno et al., 2002; Sato et al., 2008), or modulation of various ion channels (Viard et al., 2000; Kathöfer et al., 2003; Scherer et al., 2007; Hristov et al., 2008), but most of these additional pathways appear restricted to some cell types. Many β_3_-adrenoceptor ligands exhibit biased agonism, i.e., preferentially stimulate one signaling pathway relative to another (Evans et al., 2010). For instance, CL 316,243 preferentially activates cAMP formation (Evans et al., 2010), whereas L 748,337 and SR 59,230 are antagonists or weak partial agonists for cAMP formation but much more efficacious agonists for ERK and p38 phosphorylation (Hutchinson et al., 2005; Sato et al., 2007; Sato et al., 2008). At least in some cell types, β_3_-adrenoceptors can additionally couple to G_i_ proteins, and this may mediate their coupling to ERK phosphorylation (Gerhardt et al., 1999; Soeder et al., 1999; Cao et al., 2000; Sato et al., 2008). Whether biased agonists at β_3_-adrenoceptors also cause desensitization of cAMP formation and/or whether agonists also desensitize pathways other than cAMP formation has not been reported. Desensitization of non-canonical signaling has until now been explored for only one GPCR, the µ opioid receptor primarily coupling to G_i_ (Raehal et al., 2011). Of note, it has been questioned whether formation of cAMP mediates relaxation of urinary bladder smooth muscle by β-adrenoceptor agonists (Frazier et al., 2005; Uchida et al., 2005) and an involvement of alternative signaling pathways has been postulated (Frazier et al., 2011).

Therefore, the present study was designed to compare desensitization patterns of canonical (cAMP formation) and alternative signaling (ERK phosphorylation) of β_3_-adrenoceptors. Secondary aims were to compare desensitizing properties of a panel of six full and partial including biased agonists. For this purpose, we have used HEK cells stably transfected with human β_3_-adrenoceptors at presumed physiological density, because this is the model in which agonist-induced desensitization of cAMP formation has been shown most consistently (Chaudhry and Granneman, 1994; Vrydag et al., 2009; Michel-Reher and Michel, 2013). The primary aim could not be addressed because we found during the study that the ERK phosphorylation in response to stimulation with β-adrenoceptor agonists was insufficiently robust to allow testing of its desensitization (see **Online Supplement**). Therefore, this manuscript focusses on our secondary aim, the comparison of desensitization of cAMP accumulation by full, partial and biased agonists.

## Methods

### Materials

Dulbecco’s modified Eagle medium (DMEM), F12 nutrient mixture, and geneticin were from Gibco (Thermo Fisher Scientific, Waltham, MA, USA), Hank’s balanced salt solution (HBSS) was either from Gibco or from Sigma Aldrich (Munich, Germany). Bovine serum albumin (BSA), fetal calf serum (FCS), enzyme-free cell dissociation solution, penicillin/streptomycin, HEPES, isobutylmethylxanthine (IBMX), phosphate-buffered saline (PBS), 4-[[(hexylamino)carbonyl]amino]-*N*-[4-[2-[[(2*S*)-2-hydroxy-3-(4-hydroxyphenoxy)propyl]amino]ethyl]phenyl]-benzenesulfonamide (L755,507), CL316,243 (disodium 5-[(2R)-2-[[(2R)-2-(3-chlorophenyl)-2-hydroxyethyl]amino]propyl]-1,3-benzodioxole-2,2-dicarboxylate hydrate), SR59,230A ((2*S*)-1-(2-ethylphenoxy)-3-{[(1*S*)-1,2,3, 4-tetrahydronaphthalen-1-yl]amino}propan-2-ol), trypsin-ethylenediaminetetraacetic acid (EDTA) solution, forskolin, and isoprenaline bitartrate were from Sigma-Aldrich. Dimethyl sulfoxide (DMSO) was from PanReac AppliChem (Darmstadt, Germany). L748,337 (N-[[3-[(2S)-2-hydroxy-3-[[2-[4-[(phenylsulfonyl)amino]phenyl]ethyl] amino]propoxy] phenyl]methyl]-acetamide) and pertussis toxin (PTX) were obtained from Tocris Bioscience (*via* Bio-Techne, Wiesbaden-Nordenstadt, Germany). Solabegron HCl was provided by Velicept Therapeutics, Inc. (Malvern, PA, USA). The AlphaScreen cAMP assay kit and the AlphaPlate 384 were obtained from PerkinElmer (Waltham, MA, USA).

The following stock solutions were prepared and stored in aliquots at −20°C: isoprenaline bitartrate dissolved at 10 mM in distilled water (+1 drop HCl 10 mM in 1–2 mL); solabegron dissolved at 10 mM in 50% DMSO/50% distilled water; CL 316,243 dissolved at 10 mM in distilled water; L 755,507, L 748,337, and SR 59,230 dissolved at 10 mM in DMSO; forskolin dissolved at 50 mM in DMSO. PTX was dissolved at 100 μg/ml in distilled water and was stored at 4°C.

### Cell Culture

Cell culture was performed as previously described (Michel-Reher and Michel, 2013) with minor modifications. Briefly, HEK cells transfected with human β₃-adrenoceptors (Vrydag et al., 2009) were grown and passaged in an atmosphere of 5% CO₂/95% air at 37°C in DMEM/F12 supplemented with 10% FCS and penicillin (100 units/ml) and streptomycin (100 µg/ml). To maintain selection pressure, geneticin (400 µg/ml) was added to all growing cells but was not present during the experiments. As shown in our previous studies, our HEK cells do not exhibit quantifiable cAMP responses to stimulation with isoprenaline in the absence of transfection with β₃-adrenoceptors (Michel-Reher and Michel, 2013), indicating little if any expression of any subtype of functional β-adrenoceptors in the absence of transfection. In some experiments, cells were cultured for 24 h in the presence of PTX (100 ng/ml), a condition we have previously shown to be effective in HEK cells (Schmidt et al., 1995). For the desensitization experiments, cells were cultured for 24 h in serum-free medium in the presence of vehicle or indicated ligand concentrations. Based on testing in regular intervals by PCR, all experiments were performed in cells without mycoplasma contamination.

### Signal Transduction Assays

The basic experimental design and assay protocols for cAMP accumulation and its desensitization are based on Michel-Reher and Michel (2013) but now have been performed with a different cAMP assay kit according to manufacturer’s instructions. Briefly, acceptor beads and cells (625 cells/well) were added in a total volume of 5 μl/well in a 384 well OptiPlate, followed by addition of indicated concentrations of agonists (5 μl/well) and incubation for 30 min at room temperature. Finally, biotin-cAMP and streptavidin donor bead detection mix (15 μl/well) was added and incubated for 20 h at room temperature. All pipetting steps were done under subdued lighting and all incubations upon covering the plates with Topseal to avoid bleaching and evaporation. Detection was performed using the EnSpire Multimode plate reader (PerkinElmer).

### Data Analysis

Based on the standard curve, we calculated absolute amounts of accumulated cAMP per well of 25 μl within each experiment as mean of triplicate measurements per data point. Our experimental conditions were chosen to maximize chances of obtaining stimulated cAMP accumulation in the close to linear part of the standard curve. This resulted in most basal values being outside the standard curve. Therefore, we do not report basal values, and stimulated values are shown without subtraction of basal values. Similarly, the cAMP response to the lowest ligand concentration in the concentration-response experiments (1 nM) fell outside the range of the standard curve in many cases; to avoid skewed distribution and associated bias by only including values within the standard curve, the values obtained with 1 nM of ligand were excluded from the graphic representation of pooled data but not from curve fitting for individual experiments. Results from experiments were only eliminated from the analysis if the entire experiment was considered to have failed on technical grounds, e.g., if isoprenaline did not cause a measurable response in cells pretreated with vehicle.

We did not apply power calculations to determine sample size because we do not know which effect size (degree of desensitization) may be biologically meaningful. Rather, we pre-specified a sample size of n = 8 for all cAMP experiments (by mistake, effects of pretreatment with isoprenaline on concentration-response curves have been tested with n = 10). This was done based on our previous studies (Vrydag et al., 2009; Michel-Reher and Michel, 2013), in which a 24-h treatment with 10 µM isoprenaline reduced cAMP accumulation by about 70%, and this was consistently observed with n = 5–7.

Efficacy relative to the reference agonist isoprenaline was calculated within each experiment and is presented as mean with CI. Concentration-response curves within an experiment were analyzed by fitting a sigmoidal function to the experimental data to estimate E_max_ and pEC_50_. Because maximum cAMP accumulation in the absence of pretreatments exhibited considerable inter-day variability as also observed in our previous studies using the same cells (Vrydag et al., 2009; Michel-Reher and Michel, 2013) and E_max_ values were considerably closer to normal distribution upon log transformation, E_max_ data within a given group are shown as geometric means with CI. All other data are shown as arithmetic means with CI. Treatment effects on E_max_ were expressed as % of paired control, whereas those on pEC_50_ as paired difference relative to control.

For each β-adrenoceptor ligand, our study tested the following pre-specified null hypotheses:

- Pretreatment with PTX does not affect ligand-induced cAMP accumulation.
- Pretreatment with ligand does not affect cAMP accumulation in response to isoprenaline or forskolin.
- Pretreatment with ligand does not affect E_max_ or pEC_50_ of freshly added ligand.

To test these pre-specified null hypotheses, E_max_ and pEC_50_ data upon pretreatment with PTX or ligand were expressed as % of matched control values and as paired difference, respectively, each as means with CI. The null hypotheses were rejected if CI did not include 100% for E_max_ or 0 for shift of pEC_50_. All other experiments described here were performed in an explorative fashion and, accordingly, hypothesis-testing statistical analysis was not applied. All curve fitting procedures and statistical analyses were performed by the Prism program (version 7.0, GraphPad Software, San Diego, CA, USA).

## Results

**Figure 1:**
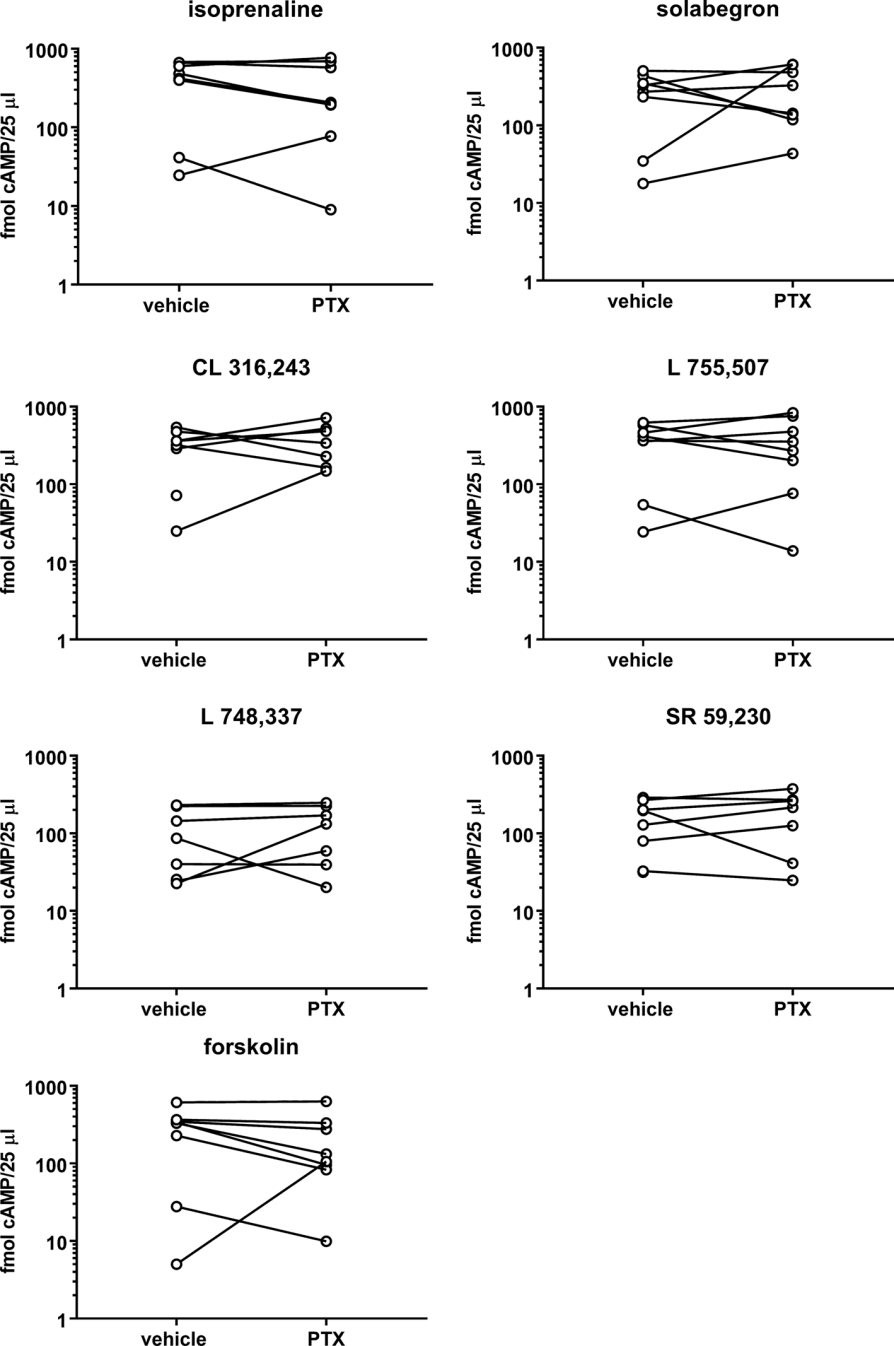
Effects of six β-adrenoceptor ligands (10 µM each) and forskolin (100 µM) on cAMP accumulation. Stimulations were performed after 24 h in the absence (vehicle) or presence of PTX (100 ng/ml). Each data point represents a single experiment and data in the absence and presence of PTX within an experiment are connected by a line. Geometric means with confidence intervals of cAMP accumulation by the six ligands and forskolin in the absence of PTX were 262 (87; 793), 178 (62; 505), 221 (90; 543), 243 (89; 665), 66 (28; 153), 116 (55; 244), and 152 (68; 608) fmol/25 µl, respectively. Following pretreatment with PTX geometric means were 190 (56; 645), 222 (101; 490), 320 (182; 562), 223 (72; 696), 93 (38; 225), 133 (51; 347), and 126 (44; 358) fmol/25 µl, respectively.

Our first experiments were designed to test the pre-specified null hypothesis that PTX does not affect cAMP responses to any of the stimulators. The comparison of the six β-adrenoceptor ligands (10 µM) in the absence of PTX indicated stimulation of cAMP accumulation with a numeric rank order of isoprenaline ≥ L 755,507 ≥ CL 316,243 > solabegron > SR 59,230 > L 748,337 (mean effect relative to isoprenaline with 95% confidence intervals (CI): 0.95 [0.75; 1.15], 0.91 [0.58; 1.259], 0.70 [0.55; 0.85], 0.53 [0.22; 0.84], and 0.36 [0.12; 0.61], respectively; **Figure 1**). cAMP responses in cells pretreated with PTX relative to vehicle treated cells were (mean % with CI): isoprenaline: 99 [20; 178], solabegron: 316 [−174; 806], CL 316,243: 182 [4; 359], L 755,507: 121 [43; 200], L 748,337: 183 [8; 3589 SR 59,230: 112 [64; 160], and forskolin: 315 [−290; 919]. Considering the wide CIs, we also performed paired, two-tailed t-tests of log-normalized cAMP values (*post hoc* analysis), which also failed to indicate a statistically significant effect of PTX. Thus, the null hypothesis was not rejected for any of the stimulators, i.e., an effect of PTX on cAMP accumulation was not shown.

**Figure 2:**
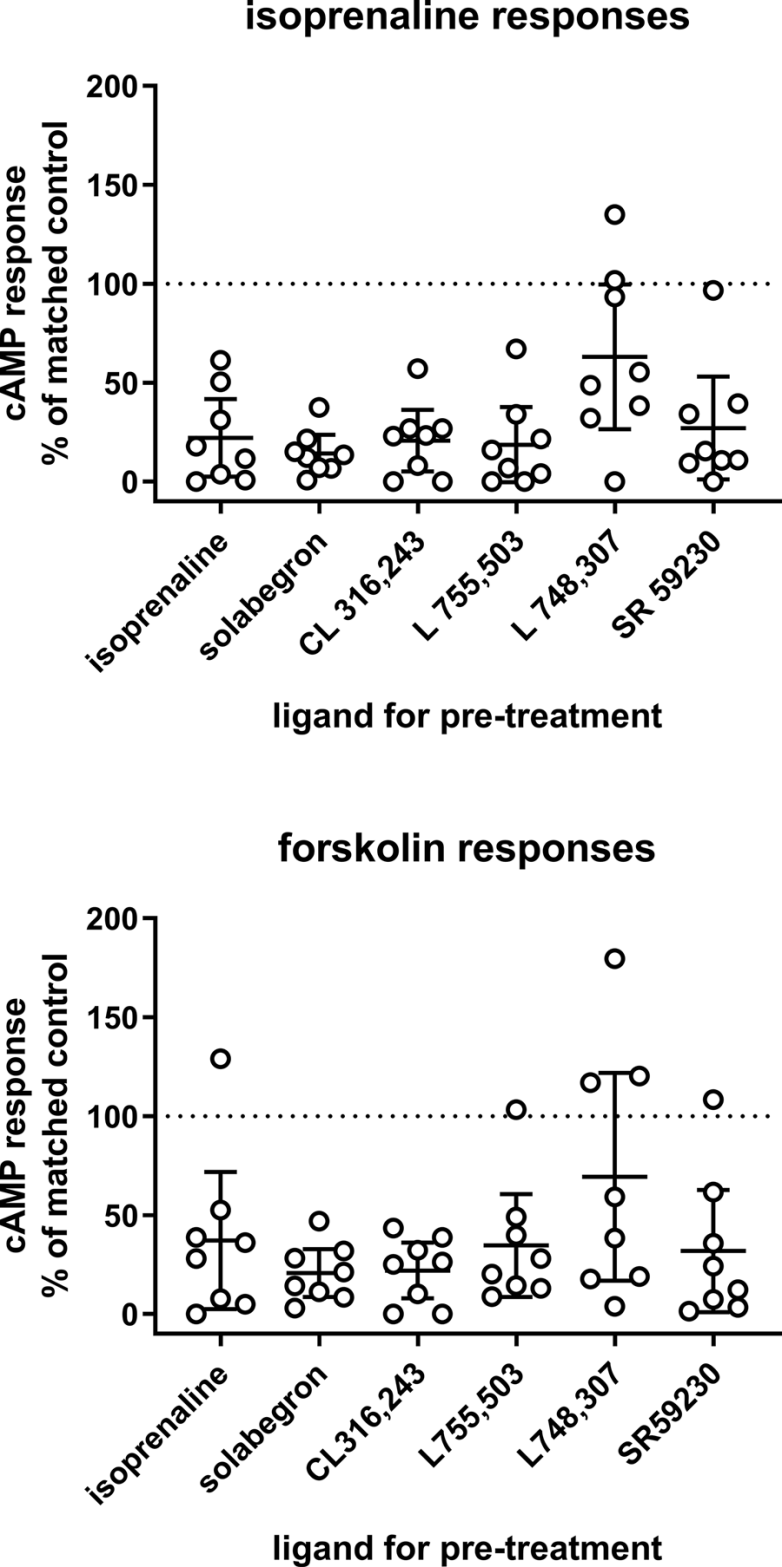
Effects of a 24 pretreatment with various β-adrenoceptor ligands (10 µM each; indicated on *x*-axis) on cAMP accumulation in response to freshly added isoprenaline (10 µM; upper panel) or forskolin (100 µM; lower panel). Data are expressed as % of paired isoprenaline or forskolin response in cells pretreated with vehicle. To increase robustness of the calculations, we included two triplicates of cells pretreated with vehicle (definition of non-desensitized response) in each experiment and set their average as 100%. In cells pretreated with vehicle, cAMP responses to isoprenaline (geometric means with CI) were 156 (76; 317) fmol/25 µl and to forskolin 232 (105; 501) fmol/25 µl. Each data point represents one experiment and horizontal line with error bar represents mean with CI. The horizontal dotted line indicates 100% of control, i.e., absence of desensitization.

We then tested the null hypothesis that pretreatment with β-adrenoceptor ligands (10 µM for approximately 24 h) did not affect cAMP accumulation stimulated by freshly added isoprenaline (10 µM) or forskolin (100 µM). To enhance robustness of the findings, we included two triplicates of data with vehicle pretreatment; despite the large variability of cAMP responses between experiments, the responses within an experiment were quite similar for both triplicates (Pearson correlation coefficient r 0.8582 and 0.8424 for stimulation by isoprenaline and forskolin, respectively). Pretreatment with all six ligands reduced cAMP responses to isoprenaline to some extent (none of the confidence intervals including 100% of vehicle control, i.e., null hypothesis rejected in all cases); mean responses ranged from 15% to 37% of vehicle control for most ligands but were 63% of control for L 748,337 (**Figure 2**). Similarly, pretreatments with most ligands reduced forskolin responses to 21–37% of control (**Figure 2**; null hypothesis rejected) but were 69% of control for L 748,337 (**Figure 2**; null hypothesis not rejected).

**Figure 3:**
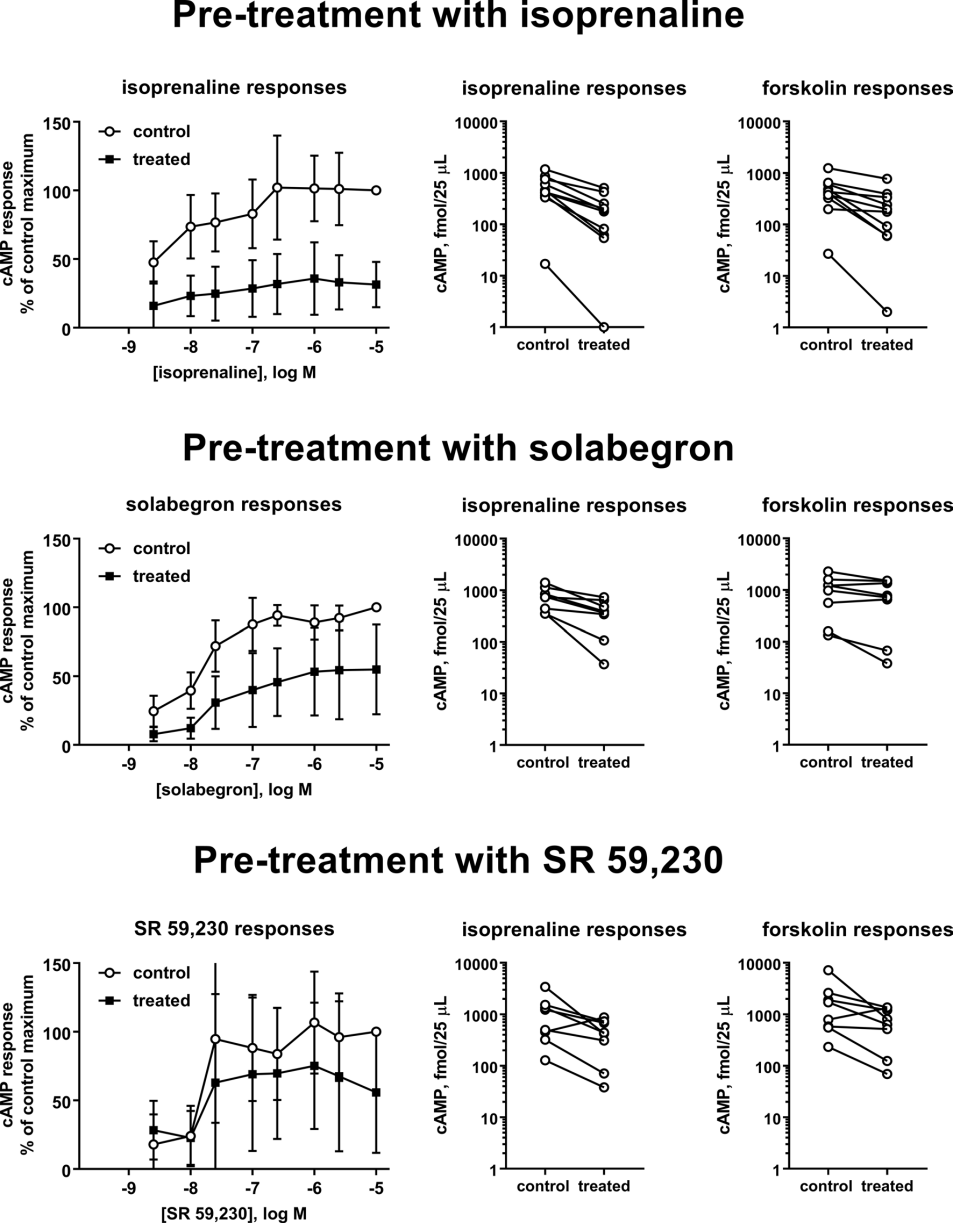
Effects of a 24 pretreatment with vehicle (control) or isoprenaline (upper panels), solabegron (middle panels), and SR 59,230 (lower panels; 10 µM each) on cAMP accumulation in response to freshly added agonist (left panels), 10 µM isoprenaline (middle panels), or 100 µM forskolin (right panels). Data from the concentration response curves are expressed as % of the response to 10 µM of the agonist in control cells. Paired isoprenaline or forskolin response in cells pretreated with vehicle. Each data point represents one experiment and data from the same experimental day are connected by lines. A quantitative analysis of the data is shown in **Tables 1** and **2**. The graphical depiction of the concentration-response curves excludes data obtained with 1 nM of ligand because, similar to basal values, many were outside the range of the standard curve and the remaining data points skewed the apparent means for this ligand concentration to higher values.

Thereafter, we selected three ligands for a more detailed analysis, isoprenaline (full agonist), solabegron (strong partial agonist), or SR 59,230 (weaker partial agonist for cAMP formation, biased agonist). We pretreated cells with 10 µM of each ligand and determined cAMP accumulation in response to a full concentration-response curve of freshly added ligand (**Figure 3**; **Table 1**). Pretreatment with isoprenaline, solabegron, and SR 59,230 reduced E_max_ of freshly added ligand to 33%, 56%, and 69% of matched control, respectively (CI not including 100% for isoprenaline and solabegron, i.e., null hypotheses rejected). Paired differences in pEC_50_ were −0.60, −0.42, and −0.03, respectively (CI not spanning 0 for solabegron, i.e., null hypothesis rejected). Of note, the potency comparison for isoprenaline is based on only 4 of 10 experiments, mostly because calculated potency was greater than the lowest tested agonist concentration in several cases, not representing a valid estimate; this left little statistical power to detect a change in pEC_50_. As part of the same experimental series, we also tested effects of the pretreatments on cAMP responses to freshly added isoprenaline (10 µM) and forskolin (100 µM). All three pretreatments numerically reduced responses to both freshly added compounds. The apparent effect sizes were largest for pretreatment with isoprenaline and for each pretreatment tended to be larger for freshly added isoprenaline than for freshly added forskolin (**Table 2**).

**Table 1:** Effect of a 24 h pretreatment with 10 µM each of isoprenaline, solabegron or SR 59,230 on potency and efficacy of freshly added ligand.

E_max_			pEC_50_		
Control, fmol/25 µl	Treated, fmol/25 µl	Treated, % of control	Control, –log M	Treated, –log M	Paired difference, –log M
Pretreatment with isoprenaline					
385 (178; 834)	79 (11; 594)	33 (18; 49)	7.35 (6.52; 8.18)	7.25 (6.11; 8.39)	−0.60 (−3.34; 2.14)
Pretreatment with solabegron					
598 (273; 1310)	258 (59; 1131)	56 (28; 85)	7.77 (7.55; 7.98)	7.35 (7.17; 7.53)	−0.42 (−0.62; −0.21)
Pretreatment with SR 59,230					
355 (200; 631)	205 (94; 445)	69 (34; 104)	7.58 (7.04; 8.13)	7.56 (7.06; 8.05)	−0.03 (−0.62; 0.57)

**Figure 4:**
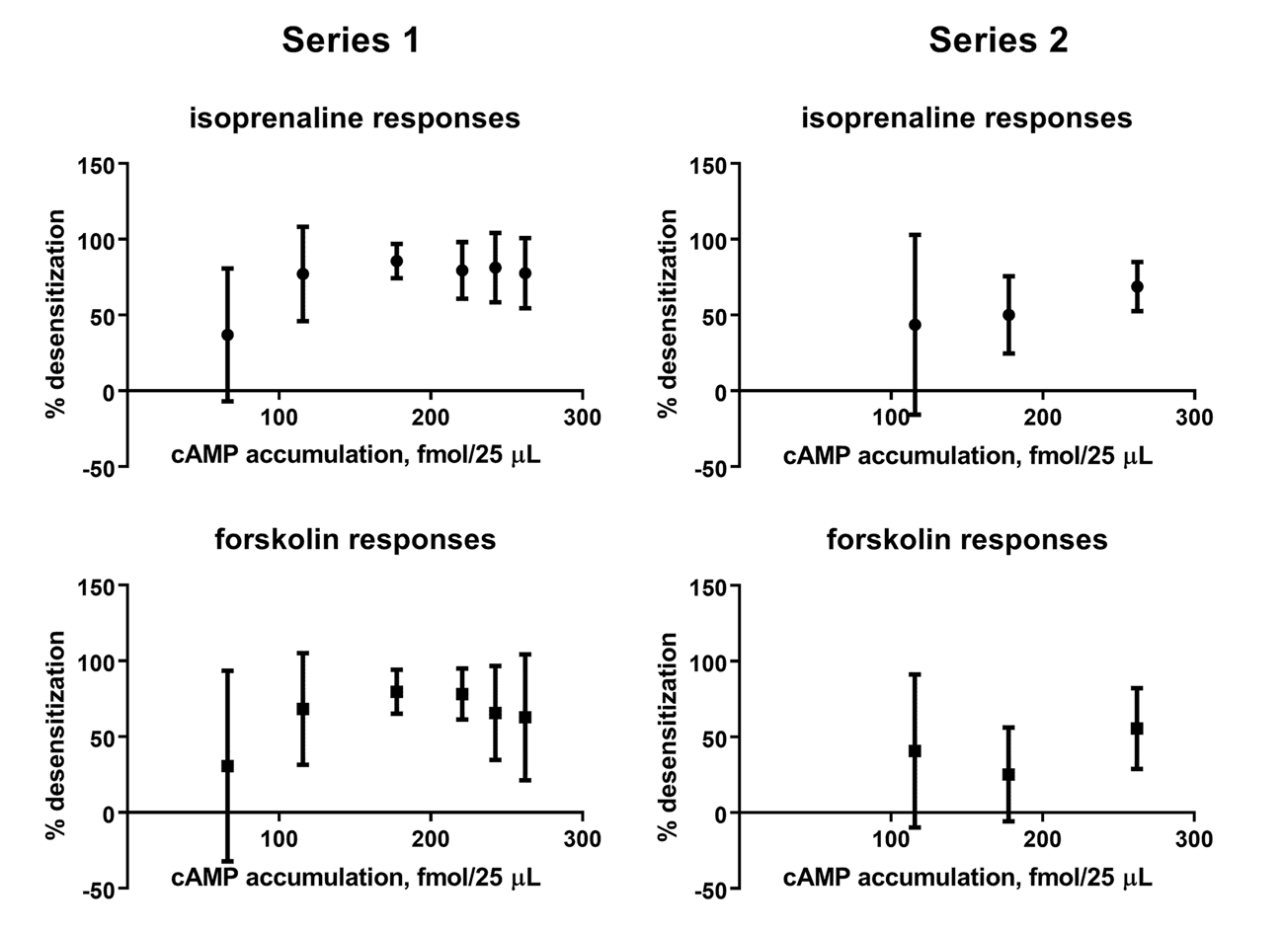
Comparison of effects on cAMP accumulation (geometric means of fmol/25 µl; see **Figure 1**) and degree of desensitization of cAMP accumulation in response to freshly added isoprenaline or forskolin [mean ± SD of 100—% response; see **Figures 2** (series 1) and **3** (series 2)] for six and three β-adrenoceptor ligands (10 µM each).

The table shows geometric means with CI for E_max_ and means with CI for pEC_50_ for control and treated cells (descriptive analysis). Treatment effects are expressed as means of treated as % of matched control with CI for E_max_ and means with CI of paired difference for pEC_50_; null hypotheses were rejected if CI for did not include 100% for E_max_ or 0 for pEC_50_. Data are based on 10 experiments for isoprenaline and eight each for solabegron and SR 59,230. Note that E_max_ could not be calculated reliably for one isoprenaline experiment (control group) and one SR 59,230 experiment (both groups); pEC_50_ could not be reliably calculated for six isoprenaline experiments (affecting four control, three treated calculations) and for two SR 59,230 experiment (both groups of a single experiment). This brings sample size for paired statistical E_max_ comparison for isoprenaline, solabegron, and SR 59,230 to nine, eight, and seven experiments, respectively, and pEC_50_ comparison to four, eight, and seven experiments, respectively. A graphical representation of the data is shown in **Figure 4**.

**Table 2:** Effect of a 24 h pretreatment with 10 µM each of isoprenaline, solabegron, or SR 59,230 on responses to freshly added isoprenaline (10 µM) or forskolin (100 µM).

Pretreatment	Isoprenaline response, % of control	Forskolin response, % of control
Isoprenaline	32 (19; 43)	44 (24; 65)
Solabegron	50 (29; 71)	75 (49; 101)
SR 59,230	56 (9; 104)	59 (17; 102)

Treatment effects are expressed as means of treated as % of matched control with CI. Null hypotheses were rejected if CI did not include 100%. Based on 10 experiments for isoprenaline and eight each for solabegron and SR 59,230. A graphical representation of the data is shown in **Figure 3**.

To explore the relationship between efficacy of the β-adrenoceptor ligands for stimulating cAMP accumulation and for causing desensitization of cAMP responses to freshly added isoprenaline or forskolin, we have plotted degree of desensitization as shown in **Figures 2** and **3** against effects on cAMP accumulation (**Figure 4**); however, there was no obvious relationship between efficacy for cAMP accumulation and ability to induce desensitization of such responses.

Despite extensive efforts, we failed to detect ERK phosphorylation responses that were sufficiently robust to study their desensitization (see **Online Supplement**). Therefore, it was not feasible to compare desensitization of cAMP and ERK phosphorylation responses.

## Discussion

The original primary aim of this study was to compare the desensitization of canonical (cAMP) and an alternative signaling pathway (ERK phosphorylation) in HEK cells stably transfected with human β_3_-adrenoceptors. As HEK cells turned out to be unsuitable to study desensitization of ERK phosphorylation (see **Online Supplement**), we primarily discuss our findings on desensitization of cAMP accumulation as induced by agonists with various degrees of efficacy including biased agonists.

### Critique of Methods

Susceptibility of β_3_-adrenoceptors to agonist-induced desensitization depends on the cell type in which the receptor is expressed (Okeke et al., 2019). The HEK cells we have generated express β_3_-adrenoceptors at presumed physiological density, i.e., about 120 fmol/mg protein upon detection with [^125^I]-iodocyanopindolol (Vrydag et al., 2009) or 550–720 fmol/mg protein upon detection with [^3^H]-L 748,337 (Michel-Reher and Michel, 2013; van Wieringen et al., 2013). It remains unclear which cell type in the urinary bladder mediates the therapeutic effects of β_3_-adrenoceptor agonists as this could involve smooth muscle, urothelial and interstitial cells within the bladder, afferent neurons, and the major pelvic ganglion (Michel and Korstanje, 2016). Therefore, it remains unclear whether HEK cells (or any other cell line) are a good model for them. However, our study has avoided artefacts due to overexpression.

As our previous work has already established the presence of agonist-induced desensitization of β_3_-adrenoceptors expressed in these HEK cells and explored the underlying molecular alterations (Vrydag et al., 2009; Michel-Reher and Michel, 2013), we have attempted to repeat only key experiments and otherwise have focused on the comparison of desensitization as induced by ligands with different degrees of efficacy including biased agonism.

We have recently reported that a 24-h treatment with the ligands used in this study can reduce number of HEK cells (Okeke et al., 2017). As our assays involve counting of cells and adjustment to desired cell density before stimulating cAMP accumulation, this reduction of cell number will not affect analysis of the present results but should be kept in mind in the overall interpretation of the data.

In line with the ongoing debate on reproducibility of scientific data (Kannt and Wieland, 2016), we have implemented various measures to promote reproducibility. Thus, all experiments were based on pre-specified sample sizes; in the absence of information, which degree of desensitization is biologically meaningful, we have chosen sample sizes to exceed those yielding consistent detection of desensitization in our previous work in the same cell line (Vrydag et al., 2009; Michel-Reher and Michel, 2013).

As our past and present experience has shown a large inter-experiment (but not intra-experiment) variability of cAMP responses in the control group, we have presented data within the respective control group as geometric means with CIs; treatment effects were expressed as percentage of paired control values, also with CIs. We also specifically identify which parts of the study were exploratory and which were based on pre-specified null-hypotheses. The latter were rejected if CIs excluded the reference value; therefore, no P values are reported. In line with recent recommendations on transparency in data reporting (Weissgerber et al., 2015), we are not showing bar graphs but scatter plots, so that each reader can appreciate the full variability within our data. In these, paired data within an experiment are connected by lines.

### Relative Efficacy and PTX-Sensitivity of Ligands

Based on reported efficacy for cAMP formation and known biased agonism properties, we have chosen a panel of six β_3_-adrenoceptor ligands to represent a wide spectrum of efficacy. Literature reports on degree of agonism for each ligand differ somewhat, possibly related to the cell type under investigation. Thus, L 755,507 was reported to be a very potent full agonist in cells with high expression density (Sato et al., 2008; Baker, 2010; Tasler et al., 2012) but efficacy was only approximately 0.5 in studies using cells lines with lower expression density (Fisher et al., 1998; Parmee et al., 1998; Kimura et al., 2000). Among ligands less frequently reported, efficacies of solabegron relative to isoprenaline for cAMP accumulation range from 0.79 to 0.89 (Uehling et al., 2006; Hicks et al., 2007); it was 0.78 for relaxation of isolated human bladder strips (Biers et al., 2006). For CL 316,243, they range from 0.5 to 0.8 for cAMP formation (Gerhardt et al., 1999; Takasu et al., 2007; Kanie et al., 2012) and were 0.41 for relaxation of human bladder (Kanie et al., 2012). Reported efficacies for two ligands commonly used as antagonists range from 0.11 to 0.43 for L 748,337 for cAMP accumulation (Sato et al., 2008; Baker, 2010) and from 0.05 to 0.36 for SR 59,230 (Hoffmann et al., 2004; Baker, 2010). Thus, the relative efficacies of the various ligands found in the present study are within the range of the reported literature values. Of note, the concentration of 10 µM used in our initial experiments was chosen to obtain close to saturation of the β_3_-adrenoceptor, particularly for the partial agonists SR 59,230 and L 748,337 that have affinities of about 117 nM (Niclauß et al., 2006) and 2 nM (van Wieringen et al., 2013), respectively. While L 748,337 and SR 59,230 have only low efficacy for cAMP accumulation, they have been identified as biased agonists efficaciously stimulating ERK phosphorylation (Hutchinson et al., 2005; Sato et al., 2007; Sato et al., 2008). These data establish that the β_3_-adrenoceptor ligands chosen for the present study cover a broad range of efficacy for cAMP accumulation and should enable studies into biased agonism.

In some cell types, β_3_-adrenoceptors not only couple to stimulation of adenylyl cyclase *via* a G_s_ protein but concomitantly also to its inhibition *via* a PTX-sensitive G_i_ protein. In those cell types, pretreatment with PTX enhances cAMP formation in response to β_3_-adrenoceptor agonists (Chaudhry et al., 1994; Soeder et al., 1999; Hutchinson et al., 2002; Sato et al., 2005; Sato et al., 2012). Our pretreatment with PTX was similar to that in those studies and had been validated to be effective in HEK cells in our previous studies (Schmidt et al., 1995); the batch of PTX used in the present study was effective in CHO cells tested around the same time (Okeke et al., 2018). Our data suggest that coupling of β_3_-adrenoceptors to G_i_ proteins in our HEK cells is too weak to allow robust enhancement of cAMP accumulation by any of the ligands in the present study. Given the numerical enhancement of cAMP accumulation by several ligands, we could not reject the null hypothesis (lack of effect of PTX) based on the pre-specified sample size but also did not provide direct evidence for a lack of G_i_ coupling. Moreover, our findings do not exclude involvement of PTX-sensitive G proteins in other signaling responses.

### Desensitization of cAMP Accumulation

β_2_-Adrenoceptors are the prototype for studying the desensitization of GPCRs. Desensitization of β_2_-adrenoceptors can occur at multiple levels of the signaling cascade including reductions of receptor expression at the mRNA and protein level (Hadcock and Malbon, 1988), decreases of G_s_ and/or increases of G_i_ protein expression (Hadcock and Malbon, 1993), a reduced expression and/or activity of adenylyl cyclase (Feldman, 1989), and an increased expression and/or function of phosphodiesterases (Ortiz et al., 2000). Previous work in transfected HEK cells has shown that agonist-induced desensitization of cAMP accumulation does not involve changes of receptor expression at the protein level or an increased expression of G_i_ protein; a decrease of G_s_ protein appears to play a minor if any role but a reduction of adenylyl cyclase activity can quantitatively fully explain the observed desensitization (Chaudhry and Granneman, 1994; Vrydag et al., 2009; Michel-Reher and Michel, 2013). Changes of mRNA expression have not been studied by us because β_3_-adrenoceptor expression in our cells is not driven by the endogenous promotor; changes in phosphodiesterase activity have not been studied because our cAMP accumulation assay is performed in the presence of phosphodiesterase inhibitors.

In previous experiments, desensitization of cAMP accumulation by isoprenaline or mirabegron consisted primarily of a reduction of E_max_ with small if any changes in pEC_50_ (Chaudhry and Granneman, 1994; Vrydag et al., 2009; Michel-Reher and Michel, 2013). Our present data confirm this observation and extend it to a strong partial agonist (solabegron) and a weak partial and biased agonist (SR 59,203).

Pretreatment with isoprenaline reduced cAMP responses to freshly added isoprenaline and forskolin to a similar extent as in previous studies (Chaudhry and Granneman, 1994; Vrydag et al., 2009; Michel-Reher and Michel, 2013), confirming the presence of both homologous and heterologous desensitization. While our previous studies had found that a reduction of adenylyl cyclase activity, measured as reduced forskolin responses, could quantitatively explain the extent of homologous desensitization of isoprenaline responses (Michel-Reher and Michel, 2013), our new data show a somewhat greater desensitization of isoprenaline than of forskolin responses for all tested ligands, indicating that desensitization may not fully be a result of reduced adenylyl cyclase responsiveness. This would be compatible with minor reductions of G_s_ expression after pretreatment with isoprenaline (Michel-Reher and Michel, 2013; Michel-Reher and Michel, 2015).

Pretreatment with solabegron, CL 316,243, L 755-503, or SR 59,230 caused a similar degree of homologous and heterologous desensitization as compared to pretreatment with isoprenaline. In contrast, pretreatment with L 748,337 caused less if any desensitization. While L 748,337 was the weakest agonist for stimulation of cAMP accumulation, a comparison of ability to stimulate cAMP accumulation with that to induce desensitization of this response did not reveal an obvious relationship between the two. This does not exclude a role for elevated intracellular cAMP as the cause for desensitization because the efficacy of SR 59,230 (0.44) may already be strong enough to induce full desensitization. This hypothesis is supported by our previous finding that a concentration of 10 nM isoprenaline (about 1/10 of EC_50_ for stimulation of cAMP accumulation) induced a more than half-maximal desensitization (Michel-Reher and Michel, 2013). Another, not mutually exclusive hypothesis is that one or more non-canonical signaling pathways may play a role in desensitization of cAMP accumulation. While we cannot exclude this possibility, we do not consider it likely considering the poor or even absent β_3_-adrenoceptor coupling to ERK phosphorylation in HEK cells (see **Online Supplement**).

### Conclusions

In conclusion, we have confirmed isoprenaline-induced desensitization of cAMP accumulation in transfected HEK cell and extend these findings by demonstrating that this can be mimicked by ligands with a wide range of efficacy including weak partial and biased agonists. These data are consistent with the idea that a minor activation of cAMP formation may be sufficient to induce full desensitization of this signaling pathway. Our data indicate that a pretreatment with PTX does not affect cAMP responses to any of the stimulators suggesting limited if any G_i_ coupling of β_3_-adrenoceptors expressed in HEK cells. All present experiments that repeat previous studies by us or others in HEK cells are highly consistent with the previous findings (Chaudhry and Granneman, 1994; Vrydag et al., 2009; Michel-Reher and Michel, 2013). However, the presence of agonist-induced β_3_-adrenoceptor desensitization may be very different in other cell types (see Introduction). For instance, only limited if any desensitization was found in rat urinary bladder strips and, in contrast to our finding in HEK cells, did not include attenuation of forskolin responses (Michel, 2014). In addition, we were unable to detect robust pERK phosphorylation in our study, which would be in line with the hypothesis that coupling to pERK formation is mediated by PTX sensitive G_i_ proteins. Of note, all previous studies exploring a role of G_i_ in coupling of β_3_-adrenoceptors to ERK phosphorylation were done in cell lines. Thus, we speculate that the lack of enhancement of cAMP formation and of detection of ERK phosphorylation in our study may be related. Therefore, we can only speculate whether HEK cells mirror effects in target tissue; studies of the role of biased agonism in desensitization may require models other than HEK cells.

## Data Availability Statement

All datasets generated for this study are included in the manuscript and/or the supplementary files.

## Author Contributions

SG and MM conceived the experiments and designed the study. KO and MM-R performed the experiments. KO, MM-R, and MM analyzed the data. KO and MM drafted the manuscript. All authors read and approved the final version of the manuscript.

## Funding

The study has been funded in part by Velicept Therapeutics.

## Conflict of Interest Statement

SG has received a consultancy honoraria in the overactive bladder field from Astellas. MCM has received a consultancy honoraria and/or research support in this field from Apogepha, Astellas and Velicept Therapeutics and is a shareholder of Velicept Therapeutics. The study had been funded in part by Velicept Therapeutics, which had been involved in discussions on the study design; it had no influence on data acquisition or analysis. Writing responsibility was solely in the hand of the authors.

The remaining authors declare that the research was conducted in the absence of any commercial or financial relationships that could be construed as a potential conflict of interest.
