# Supplementary material for: Desensitization of cAMP Accumulation via Human β3-Adrenoceptors Expressed in Human Embryonic Kidney Cells by Full, Partial, and Biased Agonists
Source: Front Pharmacol. 2019 Jun 7;10:596. doi: 10.3389/fphar.2019.00596 (PMC6590479; doi:10.3389/fphar.2019.00596)
Supplement: Supplementary file 1 [file DataSheet_1.docx]

**Online Supplement to**

**Desensitization of cAMP Accumulation via Human β_3_-Adrenoceptors Expressed in Human Embryonic Kidney Cells by Full, Partial and Biased Agonists**

Katerina Okeke^1,2^, Martina B. Michel-Reher^1^, Stavros Gravas^2^, Martin C. Michel^1^

^1^Dept. of Pharmacology, Johannes Gutenberg University, Mainz, Germany

^2^Dept. of Urology, University of Thessaly, Larissa, Greece

**Methods of ERK phosphorylation experiments**

The AlphaLisa SureFire Ultra pERK 1/2 Assay kit (Perkin Elmer, Waltham, MA, USA) recommends performing assessment of ERK phosphorylation by a two-plate method for adherently growing cells and a one-plate method for cells growing in suspension. Based on these recommendations, we initially applied the two-plate assay. However, inter-sample variability within an experiment was too large to allow robust quantification of phosphorylated ERK (pERK). As replicates of lysate from a single well consistently yielded low variability, we assume that the high variability is due to variable loss of loosely attached cells during the washing step. This was similarly observed in 96 well (recommended standard), 24 and 48 well plates and in standard and coated plates from several manufacturers. Therefore, we switched to the one-plate assay which does not involve washing steps and, accordingly, yielded less inter-sample variability within an experiment. For the one-plate assay, cells were cultured in serum-free medium for 24 h, harvested, counted and resuspended in HBSS. We then added 4 µL of suspension (5 million/mL or dilutions thereof) to each well in a 384 well Optiplate. Following incubation for two hours at 37°, we stimulated the cells by adding 4 µL vehicle or isoprenaline solution (10 µM final concentration, dissolved in HBSS supplemented with 0.1% BSA). After 5 min of stimulation, reactions were stopped by adding 2 µL lysis buffer, followed by placing the plate on a rotary shaker for 10 min. Thereafter, Acceptor beads mix (5 μL) was added followed by another 10 min of incubation at room temperature, and then addition of the streptavidin mix (5 μL) under subdued lighting and covered by TopSeal. After another 2 min on the rotary shaker, the plates were incubated at room temperature for approximately 20 h under coverage with TopSeal and followed by detection using the Enspire Multimode plate reader (Perkin Elmer). This consistently yielded quadruplicates with low inter-sample variability.

In contrast, our pERK assay does not include a standard curve and only allows quantification of signals relative to basal values within the same experiment. Therefore, pERK data are presented as % of matched basal with each data point being the mean of a quadruplicate measurement (see Results).

As we had not previously done ERK activation studies with β_3_-adrenoceptors in HEK cells, we had no basis for sample size determination for ERK phosphorylation; after having tried numerous assay conditions with the two-plate and the one-plate method, we stopped the assessment of ERK phosphorylation after n = 5 in the final chosen protocol (see above) because of obvious futility.

**Results of ERK phosphorylation experiments**

We have tested a range of experimental conditions to explore effects of isoprenaline on ERK phosphorylation; these included using the two-plate and one-plate method as described by the manufacturer, cell culture plates with and without coating from several manufacturers, various stimulation times and various cell numbers in the assay. None of them yielded robustly detectable ERK phosphorylation. Based on these pilot experiments, we considered using 10 µM isoprenaline for 5 min in the one-plate assay (see Methods) as the most promising approach and have tested this in 5 experiments each using 2,500, 5,000, 10,000 and 20,000 cells/well. These experiments, in line with the various pilot experiments, did not detect robust pERK stimulation by as compared to basal values (Supplemental Figure).

**Discussion of ERK phosphorylation experiments**

ERK phosphorylation is an alternative signalling pathway for β_3_-adrenoceptors detectable in some cell types such as CHO cells (Hutchinson et al., 2005; Sato et al., 2007; Sato et al., 2008) but, similar to other G_s_-coupled receptors, β_3_-adrenoceptors tend to cause only moderate ERK phosphorylation unless expressed at very high densities. Methods such as immunoblotting may be more sensitive to detect ERK phosphorylation than the AlphaLisa SureFire Ultra pERK 1/2 Assay kit used in the present study. However, the aim of the present study has not been to test whether β_3_-adrenoceptor stimulation can activate ERK but rather whether pre-treatment with agonist desensitizes this. This study aim requires a robust signal and an assay suitable for the large number of data points needed for concentration-responses curves with and without pre-treatment.

Despite intensive efforts including numerous assay variations, we were unable to detect robustly quantifiable isoprenaline-induced ERK phosphorylation in the HEK cells. Two findings indicate that this is not a false negative. Firstly, a positive control for pERK (included in the kit) was used in each experiment and always yielded a positive signal. Second, we detected β_3_-adrenoceptor-induced ERK phosphorylation using the same methods in transfected CHO cells (Okeke et al., 2018).

The lack of pERK detection in the present study may not be surprising as β_3_-adrenoceptor-induced ERK phosphorylation has been reported to be a PTX-sensitive cellular response (Gerhardt et al., 1999; Soeder et al., 1999; Cao et al., 2000; Sato et al., 2008) and our present and previous data (Michel-Reher and Michel, 2013) do not support major G_i_ coupling of β_3_-adrenoceptors expressed in HEK cells. Moreover, data obtained with β_2_-adrenoceptors in a different model suggest that cAMP-elevating agents may have inhibitors effects on ERK phosphorylation (Pon et al., 2016), raising the possibility that stimulatory and inhibitory effects may cancel each other in some cell types. Therefore, we conclude that HEK cells are unsuitable to study desensitization of ERK phosphorylation by β_3_-adrenoceptor agonism; other cell types may be required to address this question. In line with this conclusion, it has been reported that ERK phosphorylation previously observed upon stimulation with isoprenaline in HEK cells is not mediated by β- but rather by α_1A_-adrenoceptors (Copik et al., 2015). Of note, this conclusion explicitly does not rule out the existence of weak β_3_-adrenoceptor coupling to ERK phosphorylation in HEK cells. On the other hand, it should be noted that biased signalling at β_3_-adrenoceptors is not limited to ERK phosphorylation but may also involve other pathways such as p38 phosphorylation or extracellular acidification rate (Sato et al., 2007; Sato et al., 2008).

**Supplemental Figure:** Effect of 10 µM isoprenaline on pERK at different cell numbers per well. Each data point represents one experiment and is the mean in the isoprenaline quadruplicate expressed as % of the mean in basal quadruplicate. Basal values varied considerably between experiments and ranged from 4,000 to 76,000 for 2,500 cells, from 16,000 to 250,000 for 5,000 cells, from 52,000 to 490,000 for 10,000 cells, and from 126,000 to 625,000 for 20,000 cells per well. Horizontal lines are means with 95% confidence intervals; the dashed horizontal line indicates the line of identity with basal.

**References**

Cao, W., Luttrell, L.M., Medvedev, A.V., Pierce, K.L., Daniel, K.W., Dixon, T.M., et al. (2000). Direct binding of activated c-src to the ß_3_-adrenergic receptor is required for MAP kinase activation. *J Biol Chem* 275(49)**,** 38131-38134. doi: 10.1074/jbc.C000592200.

Copik, A.J., Baldys, A., Nguyen, K., Sahdeo, S., Ho, H., Kosaka, A., et al. (2015). Isoproterenol acts as a biased agonist of the alpha-1A-adrenoceptor that selectively activates the MAPK/ERK pathway. *PLoS One* 10(1)**,** e0115701. doi: 10.1371/journal.pone.0115701.

Gerhardt, C.C., Gros, J., Strosberg, A.D., and Issad, T. (1999). Stimulation of the extracellular signal-regulated kinase 1/2 pathway by human beta-3 adrenergic receptor: new pharmacological profile and mechanism of action. *Mol Pharmacol* 55(2)**,** 255-262. doi: 10.1124/mol.55.2.255.

Hutchinson, D.S., Sato, M., Evans, B.A., Christopoulos, A., and Summers, R.J. (2005). Evidence for pleiotropic signaling at the mouse ß_3_-adrenoceptor revealed by SR59230A [3-(2-ethylphenoxy)-1-[(1,S)-1,2,3,4-tetrahydronapth-1-ylamino]-2S-2-propanol oxalate]. *J Pharmacol Exp Ther* 312(3)**,** 1064-1074. doi: 10.1124/jpet.104.076901.

Michel-Reher, M.B., and Michel, M.C. (2013). Agonist-induced desensitization of human ß_3_-adrenoceptors expressed in human embryonic kidney cells. *Naunyn-Schmiedeberg's Arch Pharmacol* 386(10)**,** 843-851. doi: 10.1007/s00210-013-0891-y.

Okeke, K., Michel-Reher, M., and Michel, M.C. (2018). b_3_-Adrenoceptor desensitisation in CHO cells: comparison of cAMP and ERK signalling. *pA_2_ online* 18(1)**,** 017.

Pon, C.K., Lane, J.R., Sloan, E.K., and Halls, M.L. (2016). The β2-adrenoceptor activates a positive cAMP-calcium feedforward loop to drive breast cancer cell invasion. *FASEB J* 30(3)**,** 1144-1154. doi: 10.1096/fj.15-277798.

Sato, M., Horinouchi, T., Hutchinson, D.S., Evans, B.A., and Summers, R.J. (2007). Ligand-directed signaling at the ß_3_-adrenoceptor produced by SR59230A relative to receptor agonists. *Mol Pharmacol* 74(5)**,** 1359-1368. doi: 10.1124/mol.107.035337.

Sato, M., Hutchinson, D.S., Evans, B.A., and Summers, R.J. (2008). The ß_3_-adrenoceptor agonist 4-[[(hexylamino)carbonyl]amino]-N-[4-[2-[[(2S)-2-hydroxy-3-(4-hydroxyphenoxy)propyl]amino]ethyl]-phenyl]-benzenesulfonamide (L755507) and antagonist (S)-N-[4-[2-[[3-[3-(acetamidomethyl)phenoxy]-2-hydroxypropyl]amino]-ethyl]phenyl]benzenesulfonamide (L748337) activate different signaling pathways in Chinese hamster ovary-K1 cells stably expressing the human ß_3_-adrenoceptor. *Mol Pharmacol* 74(5)**,** 1417-1428.

Soeder, K.J., Snedder, S.K., Cao, W., della Rocca, G.J., Daniel, K.W., Luttrell, L.M., et al. (1999). The ß_3_-adrenergic receptor activates mitogen-activated protein kinase in adipocytes through a G_i_-dependent mechanism. *J Biol Chem* 274(17)**,** 12017-12022. doi: 10.1074/jbc.274.17.12017.
